# Prognostic Implications of Combined Imaging and Histologic Criteria in Squamous Cell Carcinoma with Mandibular Invasion

**DOI:** 10.3390/jcm9051335

**Published:** 2020-05-03

**Authors:** Chena Lee, Yoon Joo Choi, Kug Jin Jeon, Dong Wook Kim, Woong Nam, Hyung Jun Kim, In-Ho Cha, Sang Sun Han

**Affiliations:** 1 Department of Oral and Maxillofacial Radiology, Yonsei University College of Dentistry, 50-1 Yonsei-ro Seodaemun-gu, Seoul 03722, Korea; chenalee@yuhs.ac (C.L.); YJCHOI79@yuhs.ac (Y.J.C.); DENTJEON@yuh.ac (K.J.J.); 2 Department of Oral and Maxillofacial Surgery, Yonsei University College of Dentistry, 50-1 Yonsei-ro Seodaemun-gu, Seoul 03722, Korea; DWKIMOMFS@yuhs.ac (D.W.K.); OMSNAM@yuhs.ac (W.N.); KIMOMS@yuhs.ac (H.J.K.); CHA8764@yuhs.ac (I.-H.C.)

**Keywords:** tumor staging, diagnostic imaging, squamous cell carcinoma, oral cancers, computed tomography, magnetic resonance imaging

## Abstract

Prognosis prediction of squamous cell carcinoma (SCC) with mandibular invasion is controversial, and a more sophisticated staging system to aid prognosis could be developed with imaging characteristics of bone invasion. Imaging-feature analysis provides practical, stratified results for survival prognosis in oral SCC (OSCC) of the mandible, and imaging advances enable more detailed tumor visualization. We retrospectively evaluated significant bone-invasion features associated with poor outcomes in mandibular OSCC to assess the predictive value of staging criteria that combined imaging features and histologic grade (combined imaging–histology (IH) grade) in 65 patients (39 men, 26 women) with mandibular SCC diagnosed from 2006 to 2016. Clinicopathologic features, including T-stage and histologic grade, and prognosis were retrieved. Tumors were classified into three types by extent of mandibular invasion on pretreatment imaging studies. Moreover, we assessed the involvement of the mandibular canal. We examined the correlation of factors associated with locoregional recurrence and overall mortality. The Harrell Concordance Index (C-index) determined prognostic performance of predictors. Nineteen (29%) patients showed locoregional recurrence and 13 (20%) died. For locoregional recurrence and mortality rates, imaging-detected mandibular canal (MC) involvement is a stronger prognostic factor for recurrence (C-index = 0.61 > 0.58) and survival (C-index = 0.58 > 0.63) than histopathologically confirmed perineural invasion, as was the IH grade, especially IH Grade 3, which was significantly associated with worse locoregional recurrence (*p* < 0.02). Imaging-based staging showed higher prognostic performance than T-staging (C-index = 0.57 (recurrence), 0.60 (death)), when combined with histologic grading (C-index = 0.69 for both) or used alone (C-index = 0.63 (locoregional recurrence), 0.69 (death)). Overall survival was significantly stratified by Imaging type and IH grade. Therefore, analysis of imaging features provided more specific, practical results for survival prognosis in mandibular OSCC. Imaging advances can potentially provide detailed gross views of tumor masses to facilitate development of prognostic criteria for OSCC.

## 1. Introduction

Oral squamous cell carcinoma (OSCC) is a head and neck cancer with high recurrence and poor prognosis. Currently, there are few good prognostic factors for OSCC [1]. Despite considerable improvements in diagnostic technology over the past few decades, the five-year overall survival of OSCC has not improved significantly [2]. TNM staging, developed by the American Joint Committee on Cancer (AJCC), is generally used for the prediction of prognosis. Other factors, such as extranodal extension, pattern of invasion, and perineural and lymphovascular invasion, are considered as intermediate risk factors. Several studies have attempted to identify more deterministic prognostic factors, and the AJCC is working on a primitive expectation model of OSCC prognostic factors [3].

Tumor bone invasion and intraosseous extension are critical factors for planning surgical management of OSCC, and they affect patient prognosis [4]. However, the current tumor staging system only examines whether there is bone marrow involvement, and the extent and invasive pattern of the tumor are not specified in detail [3]. The correlation between the extent and aggressive pattern of bone marrow invasion in OSCC with prognosis has not been well studied [5,6]. This is thought to be because of the relatively low proportion of squamous cell carcinoma (SCC) invading the bone in the oral cavity. However, considering the invasive pattern as well as the fact that the surgical method for bone-invading SCC is dissimilar from that used for invading soft tissue only, careful evaluation of the extent and prognosis is necessary.

Recent studies have reported an increase in the incidence of bone-invading OSCC of up to 55%, compared to approximately 10% in previous studies [4,5,6]. Furthermore, the mandibular structure facilitates easier tumor cell invasion into the mandibular canal (MC), mental foramen, and tooth-supporting structures, such as the periodontal ligament space [7]. Therefore, the aggressive characteristics and the extent of bone invasion of the SCC on the mandible require more detailed evaluation on diagnostic imaging than the current staging system to assess their effect on patient prognosis.

This study was conducted to identify imaging features of significant bone invasion associated with poor outcomes in mandibular OSCC. Moreover, we analyzed the prognostic value of grading criteria that combine these imaging features and the histologic grade.

## 2. Materials and Methods

### 2.1. Patient Population

This retrospective study was approved by the ethics committee of Yonsei University Dental Hospital, and the requirement for informed consent was waived owing to the retrospective nature of the study. We reviewed records of 78 mandibular SCC patients who received treatment in our hospital from January 2006 to December 2016. Patients with both preoperative magnetic resonance imaging (MRI) and computed tomography (CT) images were included. Fourteen patients were excluded for the following reasons: primary means of treatment other than surgery (*n* = 9); no preoperative MRI or CT images (*n* = 3); and poor image quality due to metal artifact (*n* = 1).

Clinicopathological parameters were carefully reviewed, and patient clinical information, including sex, age, date of diagnosis, date of primary operation, last follow-up date, date of locoregional recurrence, and date of death, was collected. Data on histologic grade (cell differentiation), lymph node metastasis, perineural invasion, and lymphovascular invasion were retrieved. According to the AJCC manual, perineural invasion was defined as the presence of tumor cells within the nerve sheath, and lymphovascular invasion was defined as the presence of malignant cells within the endothelial-lined space of the lymphatic chain or vessel. The T-stage was carefully determined on the basis of criteria specified in the 8th AJCC manual [3].

### 2.2. Image Analysis

CT and MR images were used to assess tumor-imaging type and MC involvement. Localization of the soft tissue tumor was defined using MRI, whereas continuity and destruction of bone structure was determined using CT. CT images were acquired with Lightspeed (GE Healthcare, Milwaukee, WI, USA), and typical scan parameters were as follows: 120 kV; 300 mAs; rotation time, 1 s; pitch, 0.75; and collimation, 1.0 mm with axial image reconstruction. MRI was undertaken by using a head and neck coil with a 1.5-T scanner (Intera, Philips, Best, The Netherlands) and a 3.0-T scanner (Achieva and Ingenia, Phillips).

#### 2.2.1. Imaging Type Classification

All imaging assessments were conducted by two oral and maxillofacial radiologists with more than 10 and 7 years of experience in oncologic imaging who were blinded to the clinical and survival data. In case of disagreement, a consensus was reached through discussion.

**Figure 1:**
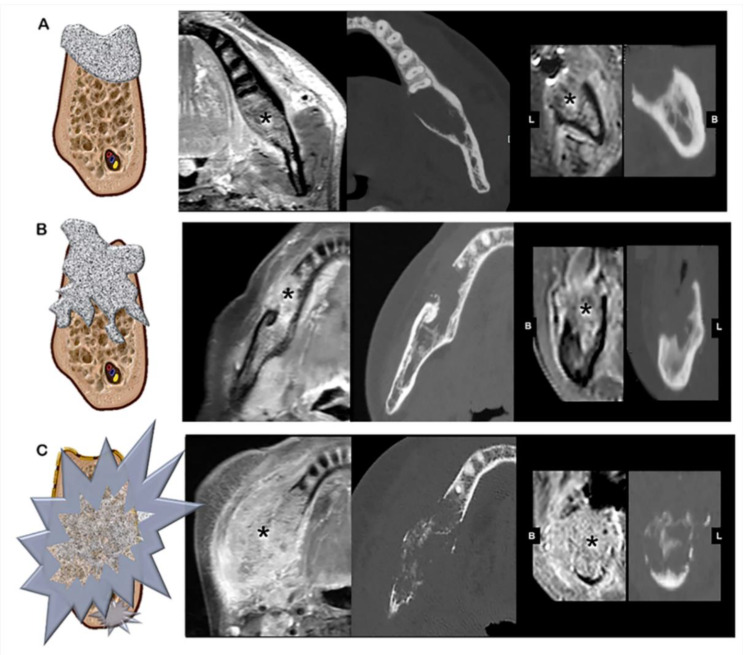
Imaging type classification. (**A**) Type I, smooth and round, with perilesional bone resorption, (**B**) Type II, rough and irregular bone margin around the lesion, and (**C**) Type III, extensive and unpredictable infiltration of the lesion into the buccal or/and lingual cortical bone. Panels on the right indicate a tumor mass on magnetic resonance imaging (MRI) and computed tomography (CT). Image sequences are, from left to right, contrast-enhanced T1-weighted MRI axial view, CT axial view, contrast-enhanced T1-weighted MRI coronal view, and CT coronal view.

The mandibular invasion pattern was classified into three types by the degree of aggressive behavior of the lesion on CT. Invasion was classified using a simplified version of the method developed in a previous study [8], as follows: Type I, contact or invasion of the bone with a smooth and remodeling margin; Type II, invasion of the underlying medullary bone with a rough and irregular margin; and Type III, extensive infiltration into the buccal and/or lingual cortical bone (Figure 1). Tumors with separate nodules from the main mass dispersed within the adjacent bone leading to bony remnant within the tumor were classified as Type III. For all imaging types, the MR images were thoroughly reviewed to check if the bone destructive area was filled with soft tissue malignant lesions.

#### 2.2.2. MC Involvement

Furthermore, MC involvement was evaluated throughout both CT and MR images. Tumors without MC involvement included distant tumors and tumors contacting the MC with intact cortication of the canal and no evidence of intracanal lesion filling. Tumors with MC involvement included tumor invasion into the canal and distinct discontinuity of the canal cortex. The continuity of the cortex was determined on CT, whereas soft tissue filling in the canal or cortex discontinuity area was determined on MR images.

### 2.3. Combined Staging Based on the Imaging Type–Histologic Grade (IH Grade)

The prognostic performance of the histologic grade in OSCC is controversial [9,10]. Therefore, we attempted to determine whether the histologic grade, which is an intermediate risk factor, can complement the prognostic ability of imaging characteristics and enhance prognostic performance.

An imaging–histology combined scoring system (IH grade) was established as Grades 1, 2, and 3, indicating mild to aggressive staging in both imaging and histology. If the histologic grade was well-differentiated and the imaging results showed Type I, the tumor was classified as Grade 1. If the tumor was either moderately or poorly differentiated and the imaging results were Type II or III, the tumor was classified as Grade 3. When the histologic grade and imaging type were not consistent, that is, the tumor had low-grade histology but aggressive behavior based on imaging results or vice versa, the classification was Grade 2.

### 2.4. Statistical Analysis

Statistical analyses were conducted in R statistical and computing software (ver. 3.4.3, www.r-project.org). The Kaplan–Meier method was used for univariate analysis. The variables analyzed included prognosis (locoregional recurrence and death) and predictors (imaging type, IH grade, and T-stage). The Harrell Concordance Index (C-index) was used to evaluate the prognostic performance of the staging parameters. Differences between C-index values were tested using the bootstrap method. Cox regression analysis was used to identify independent predictors of locoregional recurrence and death with 95% confidence intervals (CIs).

## 3. Results

General patient and tumor clinicopathologic characteristics are described in Table 1. Among the 65 patients (age, mean ± SD: 61.0 ± 12.5 years), 39 (60%) were men and 26 (40%) were women. There were 5 (8%), 10 (15%), and 50 (77%) patients with T1, T2, and T3/4 stage disease, respectively. Histopathology confirmed that 16 (24%) patients had perineural invasion. According to the imaging type, 37 (57%) cases were Type I, 23 (35%) were Type II, and four (8%) were Type III. Two cases of the Type II were difficult to determine the type, and they were fully discussed, and agreement was reached between the radiologists. Twenty-five (38%) patients presented with MC involvement (Table 1).

Within a mean follow-up period of 48 months (range: 1–155 months), 19 (29%) patients experienced locoregional recurrence, and 13 (20%) died. The prognostic performance of MC involvement was slightly higher than that of perineural invasion for both locoregional recurrence (MC involvement, C-index = 0.61; perineural invasion, C-index = 0.58) and overall death (MC involvement, C-index = 0.68; perineural invasion, C-index = 0.63). IH grade showed the strongest association with both recurrence and death. Both Imaging type and IH grade showed similar prognostic performance for death, and the C-indices were higher than that for the T-stage (IH grade, C-index = 0.69; imaging type, C-index = 0.69; T-stage, C-index = 0.60). However, the differences in prognostic performance were not significant (Table 2).

**Figure 2:**
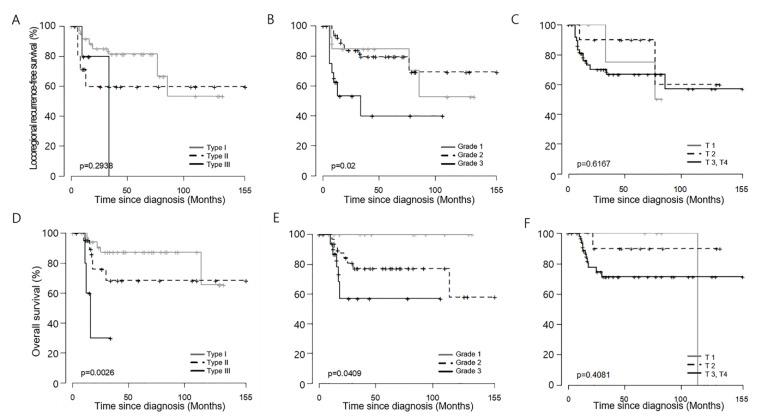
Kaplan–Meier curves of locoregional recurrence (**A**–**C**) and death (**D**–**F**) based on (**A**,**D**) Imaging type, (**B**,**E**) Imaging–Histology combined stage, and (**C**,**F**) T-stage.

On univariate analysis, MC involvement (hazard ratio (HR) = 3.47, 95% CI, 1.12–10.73, *p* = 0.031), and Imaging type III (vs. Type I, HR = 9.39, 95% CI 2.14–41.18, *p* = 0.003) were significant risk factors for death (Table 3). Kaplan–Meier curves revealed that patients with IH Grade 3 classification had significantly earlier locoregional recurrence than patients with Grade 1 and 2 tumors. Imaging type and IH grade significantly stratified overall survival (Figure 2).

## 4. Discussion

Imaging modalities play a major role in cancer diagnosis and surgical treatment planning [8,9]. However, few studies have examined associations between aggressiveness in diagnostic imaging and prognosis of OSSC, such as local failure or survival. According to the AJCC, tumors with bone marrow invasion are classified as T4, regardless of the extension of the mass into the bone [3]. It has been suggested that when SCCs invade the mandible, owing to unique structures such as the mandibular canal and tooth structures, the establishment of specific prognostic factors are required [10]. The Japanese Society of Oral Oncology defines T4 mandibular OSCC by invasion into the MC rather than merely bone marrow involvement [6]. Meanwhile, the most recent AJCC manual does not include risk factors specific for mandibular invading OSCC. Therefore, the appropriate classification of bone invasion is still debated.

In 2017, Jo et al. reported that the requisite scope of surgery can be predicted by the sclerosis of the perilesional trabecular bone [11]. This suggests that the invasion pattern of the surrounding bone may exemplify tumor characteristics. The growth of the lesion is thought to be less aggressive if the surrounding bone is remodeled with smooth margins with lesion growth, whereas when bone destruction occurs more rapidly than the remodeling, irregular boundaries can be seen [12]. If tumor proliferation occurs more rapidly than bone destruction, it can be assumed that there may be residual intralesional bone fragments. This is consistent with a histologic pattern of a poorly differentiated tumor with a cancer cell nest dispersed within the normal tissue [13]. In addition, aggressive growth causes hemorrhaging, which may turn into intralesional dystrophic calcifications.

We hypothesized that these imaging features are closely related to the overall prognosis of the patient. As a result of the study, the imaging type was a better prognostic factor than T-stage for mandibular SCC. Currently, the T-stage is the most widely used and reliable staging system. However, for mandibular SCC, this staging is rather insufficient because all of the tumor is determined as T4 when it invades the bone marrow, regardless of its extent. Thus, this study suggests the feasibility of imaging as a powerful complimentary tool for mandibular SCC staging.

Prognostic prediction was more enhanced when imaging features and histologic grade were combined in this study. The IH grade showed higher performance as a prognostic factor in mandibular SCC than T-stage. This finding is partly consistent with a previous study by Okura et al. [6], who classified 345 mandibular SCCs into three types: no bone invasion, bone marrow invasion, and MC involvement. This classification was different from that in our study, yet the most aggressive tumors, i.e., those with MC involvement, carried a higher risk of death than T3/4-stage tumors. Moreover, the researchers suggested that OSCC with bone invasion requires a more sophisticated staging system to supplement the current system, which has restricted utility.

Another interesting finding of the current study was that MC involvement on imaging showed slightly better performance as a prognostic factor of locoregional recurrence and death when compared with histologic perineural spread. Due to the fundamental limitations of imaging, microinvasion of tumor cells into the nerve was challenging to detect, although ultra-high resolution MRI is possible now. Therefore, in cases with MC involvement, large tumor size, rather than perineural invasion, might have a greater influence on prognosis.

Although, Okura et al. reported that MC involvement was a significant predictor of OS on multivariate analysis [6]. They suggested that MC involvement, rather than mere lesion size, was associated with prognosis. Unlike their multi-institutional study, the present study was conducted in only one institution with a relatively small number of cases. Thus, the influence of MC involvement on prognosis, except the tumor size effect, could not be analyzed, and we could not clearly support that MC involvement affected prognosis. Many researchers are still debating on the prognostic influence of perineural invasion in OSCC [14,15,16,17]. Further extensive research on this issue is essential.

In the present study, Kaplan–Meier curves elucidated the IH grade well stratified locoregional recurrence and overall death. IH Grade 3 showed significantly earlier locoregional recurrence than the tumors of Grade 1 and 2. Imaging alone also well described overall death according to respective type. It was less effective to clearly distinguish the prognosis for each stage of mandible invading SCC with the T-stage alone. Therefore, in the case of SCC with mandible invasion, it is carefully suggested that a more refined tumor staging in the image will aid predicting the prognosis.

This study had several limitations. As mentioned above, this study was conducted in a single institution, unlike other multicenter studies with large sample sizes. Therefore, this study had a small number of events such as recurrence and death, and therefore, it was inadequate for conducting a multivariate analysis. For the same reason, the effects of postoperative radiation therapy and surgical methods on prognosis could not be considered. These limitations should be overcome with larger study samples to establish the concrete prognostic performance of imaging type and combined grade in mandibular SCC. Based on the present study results, further multi-institutional studies with large sample sizes should be performed to consolidate a meaningful conclusion.

## 5. Conclusions

The analysis of imaging features provided more specific and practical resources for survival prognosis in OSCC. Few studies have developed imaging criteria for OSCC; therefore, this study is of importance as a cornerstone for image-guided OSCC grading. With recent advancements in imaging, which may now describe detailed as well as gross tumor features, it is important that criteria are developed to analyze the imaging features of OSCC for use as prognostic factors.

## Figures and Tables

**Table 1:** Patient clinicopathologic and imaging characteristics.

Characteristic	Value
Age (years, mean ± SD)	61.0 ± 12.5
Sex	
Female	26 (40)
Male	39 (60)
T-stage	
1	5 (8)
2	10 (15)
3	3 (5)
4	47 (72)
Lymph node metastasis	
No	46 (71)
Yes	19 (29)
Histologic grade	
Well-differentiated	23 (35)
Moderately-differentiated	36 (56)
Poorly differentiated	6 (9)
Perineural invasion	
Yes	16 (24)
No	49 (75)
Lymphovascular invasion	
Yes	11 (16)
No	54 (83)
Imaging type	
Type I	37 (57)
Type II	23 (35)
Type III	4 (8)
MC involvement	
Yes	25 (38)
No	40 (62)
Surgical procedure	
Marginal resection	17 (26)
Segmental resection	43 (66)
Hemi/Total resection	5 (8)
Postoperative radiation therapy	
Yes	32 (49)
No	33 (51)

Unless otherwise indicated, data indicate the number of patients, with percentages in parentheses. SD: standard deviation; MC: mandibular canal.

**Table 2:** Prognostic performance of predictors (Harrell’s C-index, 95% confidence interval).

	MC Involvement (Imaging)	Perineural Invasion (Histopathology)	Imaging Type	IH Grade	T-Stage
Locoregional recurrence	0.61 (0.47–0.73)	0.58 (0.50–0.68)	0.63 (0.49–0.73)	0.69 (0.57–0.80)	0.57 (0.48–0.66)
Death ^a^	0.68 (0.54–0.81)	0.63 (0.51–0.77)	0.69 (0.53–0.84)	0.69 (0.57–0.80)	0.60 (0.49–0.69)

^a^ One patient who was transferred to hospice care due to terminal stage OSCC is also included. MC: mandibular canal; OSCC: oral squamous cell carcinoma; IH: imaging–histology combined.

**Table 3:** Univariate analysis for predicting locoregional recurrence (LR) and overall survival (OS).

Variable	LR		OS	
	HR (95% CI)	*p*-Value	HR (95% CI)	*p*-Value
MC involvement (imaging)	1.84 (0.74–4.55)	0.187	3.47 (1.12–10.73)	0.031
Perineural invasion (histopathology)	1.95 (0.73–5.26)	0.187	3.14 (0.98–10.03)	0.053
Image type				
II vs. I	1.88 (0.73–4.9)	0.196	2.05 (0.59–7.10)	0.257
III vs. I	2.44 (0.51–11.58)	0.262	9.39 (2.14–41.18)	0.003
IH grade				
2 vs. 1	0.78 (0.22–2.68)	0.687	N/A ^a^	
3 vs. 1	2.85 (0.84–9.71)	0.094		
T-stage				
2 vs. 1	0.52 (0.07–3.70)	0.511	0.49 (0.03–7.85)	0.615
3/4 vs. 1	1.06 (0.24–4.70)	0.941	1.72 (0.22–13.44)	0.605

^a^ Incidence of the event was insufficient for evaluation HR: hazard ratio; CI: confidence interval; MC: mandibular canal; IH: imaging–histology combined.
